# Supplementary material for: Suppressive Role of ACVR1/ALK2 in Basal and TGFβ1-Induced Cell Migration in Pancreatic Ductal Adenocarcinoma Cells and Identification of a Self-Perpetuating Autoregulatory Loop Involving the Small GTPase RAC1b
Source: Biomedicines. 2022 Oct 20;10(10):2640. doi: 10.3390/biomedicines10102640 (PMC9599656; doi:10.3390/biomedicines10102640)
Supplement: Supplementary file 1 [file biomedicines-10-02640-s001.zip › biomedicines-1930848-supplementary.pdf]

biomedicines

Article

Suppressive Role of ACVR1/ALK2 in Basal and TGFβ1-Induced Cell Migration in Pancreatic Ductal Adenocarcinoma Cells and Identification of a Self-Perpetuating Autoregulatory Loop Involving the Small GTPase RAC1b

Hendrik Ungefroren [1,2,*], Rüdiger Braun [3], Olha Lapshyna [3], Björn Konukiewitz [2], Ulrich F. Wellner [3], Hendrik Lehnert [4] and Jens-Uwe Marquardt [1]

[1] First Department of Medicine, University Hospital Schleswig-Holstein, Campus Lübeck, D-23538 Lübeck, Germany
[2] Institute of Pathology, University Hospital Schleswig-Holstein, Campus Kiel, D-24105 Kiel, Germany
[3] Clinic for Surgery, University Hospital Schleswig-Holstein, Campus Lübeck, D-23538 Lübeck, Germany
[4] University of Salzburg, Salzburg, Austria
* Correspondence: hendrik.ungefroren@uksh.de

Abstract: Pancreatic ductal adenocarcinoma (PDAC) cells are known for their high invasive/metastatic potential, which is regulated in part by transforming growth factor β1 (TGFβ1). The involvement of at least two type I receptors, ALK5 and ALK2 that transmit downstream signals of TGFβ via different Smad proteins, SMAD2/3 and SMAD1/5, respectively, poses the issue of their relative contribution in regulating cell motility. Real-time cell migration assays revealed that selective inhibition of ALK2 by RNAi or dominant-negative interference with a kinase-dead mutant (ALK2-K233R) strongly enhanced the cells' migratory activity in the absence or presence of TGFβ1 stimulation. Ectopic ALK2-K233R expression was associated with an increase in protein levels of RAC1 and its alternatively spliced isoform, RAC1b, both of which are implicated in driving cell migration and invasion. Conversely, RNAi-mediated knockdown or CRISPR/Cas9-mediated knockout of RAC1b resulted in upregulation of the expression of ALK2, but not that of the related BMP type I receptors, ALK3 or ALK6, and elevated phosphorylation of SMAD1/5. PDAC is a heterogeneous disease encompassing tumors with different histomorphological subtypes, ranging from epithelial/classical to extremely mesenchymal. Upon treatment of various established and primary PDAC cell lines representing these subtypes with the ALK2 inhibitor LDN-193189 well-differentiated, epithelial cell lines responded with a much stronger increase in basal and TGFβ1-dependent migratory activity than poorly-differentiated, mesenchymal ones. These data show that i) ALK2 inhibits migration by suppressing RAC1/RAC1b proteins, ii) ALK2 and RAC1b act together in a self-perpetuating autoregulatory negative feedback loop to mutually control their expression, and iii) the ALK2 antimigratory function appears to be particularly crucial in protecting epithelial subtype cells from becoming invasive both spontaneously and in a TGFβ-rich tumor microenvironment.

Keywords: ALK2; ALK5; epithelial subtype; invasion; mesenchymal; phenotype; migration; pancreatic cancer; RAC1; RAC1

Supplementary Materials

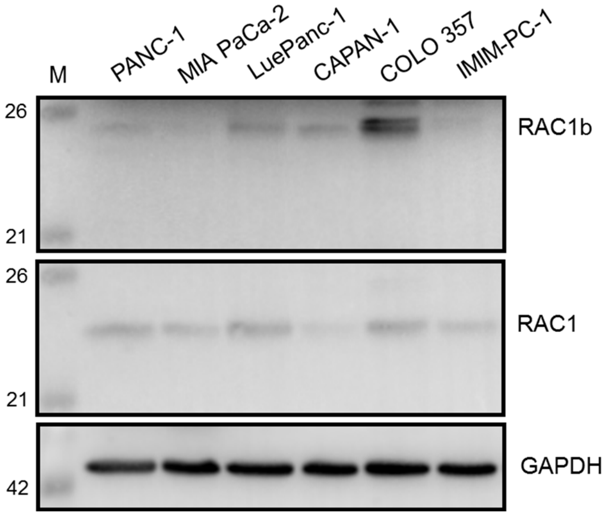

Figure S1. Immunoblot analysis of RAC1b and RAC1 in permanent PDAC cell lines (PANC-1, MIA PaCa-2, CAPAN-1, COLO 357, IMIM-PC-1) and in the primary PDAC-derived cell line LuePanc-1. The blot shown is representative of three experiments. M, molecular weight marker. The numbers to the right indicate the sizes of the marker bands in kDa.
